# Draft Genome Sequence of *Pseudarthrobacter* sp. Strain ATCC 49442 (Formerly Micrococcus luteus), a Pyridine-Degrading Bacterium

**DOI:** 10.1128/MRA.00299-20

**Published:** 2020-09-17

**Authors:** Nidhi Gupta, Kelly A. Skinner, Zarath M. Summers, Janaka N. Edirisinghe, Pamela B. Weisenhorn, José P. Faria, Christopher W. Marshall, Anukriti Sharma, Neil R. Gottel, Jack A. Gilbert, Christopher S. Henry, Edward J. O’Loughlin

**Affiliations:** a Argonne National Laboratory, Lemont, Illinois, USA; b University of Chicago, Chicago, Illinois, USA; c ExxonMobil Research and Engineering Company, Annandale, New Jersey, USA; University of Maryland School of Medicine

## Abstract

We present here the draft genome sequence of a pyridine-degrading bacterium, Micrococcus luteus ATCC 49442, which was reclassified as *Pseudarthrobacter* sp. strain ATCC 49442 based on its draft genome sequence. Its genome length is 4.98 Mbp, with 64.81% GC content.

## ANNOUNCEMENT

Micrococcus luteus ATCC 49442 was isolated from a Chalmers silt loam soil, which had not previously been exposed to pyridine, by enrichment using pyridine as a carbon, nitrogen, and energy source (1), with growth on pyridine leading to overproduction of riboflavin (2). To obtain material for sequencing, ATCC 49442 was cultured in tryptic soy broth at 30°C for 16 h, after which DNA was isolated using a DNeasy PowerSoil kit (Qiagen, catalog number 12888-50) following the manufacturer’s protocol. Two libraries were prepared for sequencing using Illumina Nextera XT paired-end library and Oxford Nanopore LSK109 kits according to each manufacturer’s protocol and sequenced on the MiSeq (8,057,766 paired-end reads; average length, 151 bp) and GridIONx5 (276,159 single-end reads; average length, 13,857 bp) sequencer platforms, respectively. Base calling was performed with Guppy v3.2.6. Demultiplexing and adaptor removal were performed with Porechop v0.2.3. We utilized the U.S. Department of Energy’s KBase system (https://kbase.us/) for assembly and genome annotation (3). During analysis, default parameters were used for all software unless otherwise specified. First, the sequence data were uploaded into KBase in FASTQ format and assembled using MaSuRCA Assembler v3.2.9 (4). The genomic sequence consisted of 4,989,141 bp on 2 contigs (64.81% GC content). The *N*_50_ value was 4,753,805 bp, and the contigs were 4,753,805 bp and 236,556 bp long. The completeness of the genome was calculated using CheckM v1.0.18 (5) and found to be 99.4% complete, with 2.81% contamination. The genome was annotated in the KBase pipeline using RAST v0.1.1 (6), and it was found that ATCC 49442 contained 4,702 coding genes, 116 noncoding repeats, and 65 noncoding RNAs (https://kbase.us/n/58065/6/).

**FIG 1:**
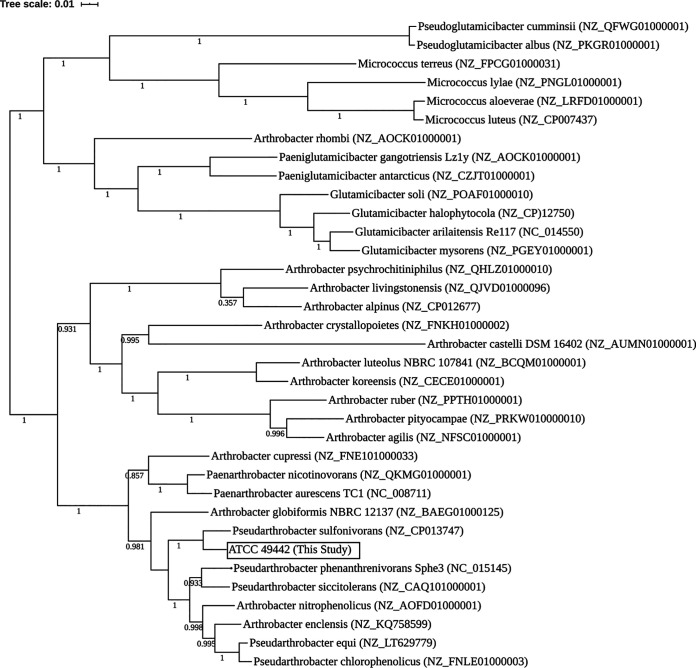
The phylogeny of ATCC 49442 in comparison to that of different species of *Arthrobacter*, *Pseudarthrobacter*, *Paenarthrobacter*, *Pseudoglutamicibacter*, *Paeniglutamicibacter*, *Glutamicibacter*, and *Micrococcus* (RefSeq accession numbers are provided in parentheses) selected from public KBase genomes. The whole genomes were aligned and the approximately maximum-likelihood tree of ATCC 49442 was constructed using the default settings of FastTree v2.1.10. Values on the tree represent the local support values for the tree nodes computed using the Shimodaira-Hasegawa (SH) test in FastTree v2.1.10.

At the time of isolation, ATCC 49442 was identified as M. luteus based on its morphological and metabolic characteristics (1). M. luteus is the type species of the genus *Micrococcus*, which is the type genus of the family *Micrococcaceae* (order *Actinomycetales*). In recent years, however, there has been considerable reorganization of genera within the *Micrococcaceae* based on phylogenetic and taxonomic analyses (7, 8). Significant reorganization of the members of the genus *Arthrobacter* has resulted in 16 different groups, including 5 novel genera, *viz*., *Pseudarthrobacter*, *Paeniglutamicibacter*, *Pseudoglutamicibacter*, *Paenarthrobacter*, and *Glutamicibacter* (9). Considering the taxonomic reorganization within the family *Micrococcaceae*, a phylogenetic tree was constructed. The tree was implemented in KBase using the “insert set of genomes into species tree” app, after first running RPS-BLAST v0.3.3 with 49 conserved marker genes based on clusters of orthologous groups (COGs) from NCBI (https://github.com/kbaseapps/SpeciesTreeBuilder/tree/master/data/cogs) against the ATCC 49442 draft genome. The RPS-BLAST alignments of marker genes from our draft genome to 34 closely related RefSeq genomes were concatenated, and the tree was then constructed. The approximately maximum-likelihood tree (Fig. 1) was built using FastTree v2.1.10 with default settings (10), based on the concatenated multiple sequence alignment of marker genes across all included genomes. This analysis indicates that the ATCC 49442 genome is deeply embedded within the genus *Pseudarthrobacter*, with maximum homology to Pseudarthrobacter sulfonivorans (Fig. 1), and we propose that this strain be classified as *Pseudarthrobacter* sp. strain ATCC 49442.

### Data availability.

The draft genome sequence of *Pseudarthrobacter* sp. strain ATCC 49442 has been deposited in GenBank under accession number JAAAXP000000000, and the raw sequencing reads are available in the Sequence Read Archive under accession numbers SRR10874394 and SRR10874393.
